# The mitogenome of the true bug *Nysius cymoides* (Insecta, Heteroptera) and the phylogeny of Lygaeoidea

**DOI:** 10.1080/23802359.2021.1951139

**Published:** 2021-07-15

**Authors:** Antonio Carapelli, Claudia Brunetti, Claudio Cucini, Elena Cardaioli, Abir Soltani, Moez Amri, Jouda Mediouni Ben Jemâa, Pietro Paolo Fanciulli, Francesco Nardi

**Affiliations:** a Department of Life Sciences, University of Siena, Siena, Italy; b Faculty of Sciences of Bizerte, Carthage University, Tunis, Tunisia; c LR11INRAT06 Laboratory of Biotechnology Applied to Agriculture, National Agricultural Research Institute of Tunisia (INRAT), Carthage University, Tunis, Tunisia; d AgroBioSciences (AgBS), Mohammed VI Polytechnic University (UM6P), Ben Guerir, Morocco

**Keywords:** Pest insects, Lygaeoidea, mitogenomics, phylogeny

## Abstract

The complete mitochondrial genome of the true bug (Homoptera) *Nysius cymoides* (Spinola, 1837) is herein described and used for phylogenetic comparison with other species of Lygaeoidea. The mtDNA has a gene order and other molecular features typically observed in hexapods, and a long A + T-rich region, due to the occurrence of several repeat units. The phylogenetic analyses support the monophyly of all families except Rhyparochromidae.

Lygaeoidea (Hemiptera: Heteroptera) is a predominantly phytophagous insect superfamily of the infraorder Pentatomomorpha that is economically important as its members exploit host plant resources. The phylogenetic relationships among the 15 families of the taxon are debated on both morphological and molecular grounds (Yao et al. 2012; Liu et al. 2019). Thus, we have applied Next generation sequencing techniques to obtain the complete mitogenome (mtDNA) of *Nysius cymoides*, an agricultural pest of susceptible crop species in the Middle East and in Europe, and an emerging pest for northern Africa, in order to assess the internal relationships of the group. Samples were collected in Le Kef (36°04'36"N, 09°01'21"E) the 21th of June 2018 on chili pepper (*Capsicum annum* L.) (Solanaceae). Voucher specimens (sampling codes: NCY-LKEF1-10) are deposited in the Department of Life Sciences, University of Siena, Italy (Antonio Carapelli, antonio.carapelli@unisi.it). Total genomic extraction was performed using the QIAmp^®^ UCP DNA kit. Libraries were prepared using the TruSeq DNA Nano kit (Illumina, San Diego, CA) with 350 bp insert length and 151 bp paired-end sequences were obtained on a HISeq 2500 platform (Illumina, San Diego, CA) at Macrogen Europe. Resultant reads were de novo assembled as in Nardi et al. (2020). Briefly, the cox1 gene was employed as seed in the NOVOPlasty v. 3.8.3 (Dierckxsens et al. 2017) assembling method with *k* = 39–129. The resulting assemblies were checked against a MEGAHIT assembly (Li et al. 2015) and manually curated. The final mtDNA sequence was automatically annotated using MITOS (Bernt et al. 2013) and automatic annotations were revised manually by comparison with related species.

The mtDNA of *N. cymoides* (GenBank accession number: MW291653) is a circular molecule of 16,301 bp. and conforms with the general molecular features observed for insects, in having 13 protein-encoding genes (PCGs), 22 transfer RNA genes (tRNAs), 2 ribosomal RNA genes (rRNAs) (encoding for *rrnL* and *rrnS*), and a putative A + T-rich “control” region (Cucini et al. 2021). The gene order is identical to that considered ancestral for Pancrustacea, with a set of tRNA-encoding genes that can be folded in the canonical cloverleaf secondary structure, except that for Serine (*trnSgcu*) that lack the DHU arm as observed in most Metazoa’s mtDNA (Comandi et al. 2009).

The *N. cymoides* mtDNA (all J-strand) shows a substantial nucleotide bias toward a higher A and T content (AT% = 77.1; A% = 43.6; C% = 13.3; G% = 9.6%), with positive AT- and CG-skew (0,1320 and 0,1614, respectively) for the J-strand. Most of PCGs (*atp6, atp8, cox2, cox3, cob* and *nad2-4*) starts with canonical codons (either ATA or ATG) encoding for Methionine; whereas two genes (*nad1* and *nad6*) use ATT (encoding for Isoleucine), and two others (*cox1* and *nad4L*) use TTG (encoding for Leucine). Stop codons are either complete codons (TAA for *atp6, nad1, nad4* and *nad4L*) or truncated codons (TA– for *atp6, cox3, nad2, nad5* and *nad6*; T–– for *cox1, cox2, cob* and *nad3*).

As for the A + T-rich region, it accounts for a total 1746 bp, the sum of 766 bp, plus an almost identical DNA fragment of 140 bp, presumably repeated 7-times.

The phylogenetic analysis was performed using a Bayes inference method (MrBayes, v. 3.2.7a; Ronquist et al. 2012) applied to a data set inclusive of all 13 PCGs of 13 Lygaeoidea species, plus two outgroups. The resulting tree (Figure 1) is similar to that obtained by Cao et al. (2020) and highlights the monophyly of families Lygaeidae, Bertydae, Malcidae, but not of Rhyparochromidae. One Rhyparochromidae species (*Neolethaeus assamensis*) is the basalmost taxon to Colobathristidae plus the remaining ingroups. As expected, *Nysius cymoides* clusters with the congeneric species *N. plebeius*, with *N. fuscovittatus* their sister group. In detail, deep and more recent nodes of the tree are more supported internal, probably suggesting that the enclosure of additional key taxa is necessary to unravel the evolutionary relationships of Lygaeoidea.

**Figure 1.**
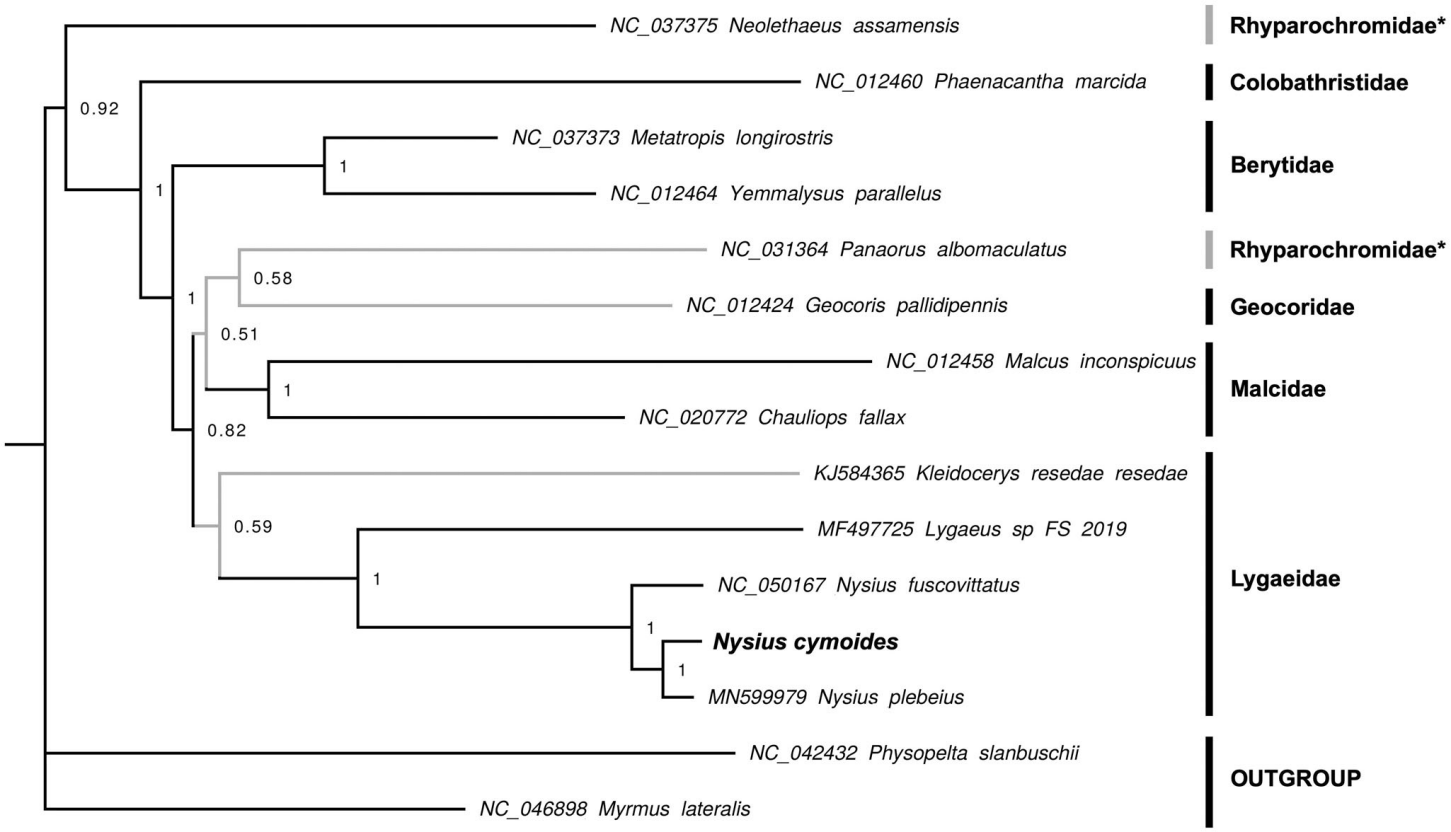
Bayesian tree based on 13 PCGs of 13 Lygaeoidea (values of posterior probabilities reported on nodes). In gray, not supported lineages.

## Data Availability

The genome sequence data that support the findings of this study are openly available in GenBank of NCBI at https://www.ncbi.nlm.nih.gov/nuccore/MW291653.1/, under the accession no. MW291653. The associated BioProject, SRA, and Bio-Sample numbers are PRJNA673074, SRS7612484, and SAMN16587686, respectively.
